# Repurposing the Kinase Inhibitor Mavelertinib for Giardiasis Therapy

**DOI:** 10.1128/aac.00017-22

**Published:** 2022-06-15

**Authors:** Samantha A. Michaels, Matthew A. Hulverson, Grant R. Whitman, Linh T. Tran, Ryan Choi, Erkang Fan, Case W. McNamara, Melissa S. Love, Kayode K. Ojo

**Affiliations:** a Department of Medicine, Division of Allergy and Infectious Diseases, Center for Emerging and Reemerging Infectious Diseases (CERID), University of Washington, Seattle, Washington, USA; b Department of Biochemistry, University of Washington, Seattle, Washington, USA; c Calibr, Division of the Scripps Research Institute, La Jolla, California, USA

**Keywords:** ReFRAME library, *Giardia lamblia* strains, EGFR tyrosine kinase inhibitors

## Abstract

A phenotypic screen of the ReFRAME compound library was performed to identify cell-active inhibitors that could be developed as therapeutics for giardiasis. A primary screen against Giardia lamblia GS clone H7 identified 85 cell-active compounds at a hit rate of 0.72%. A cytotoxicity counterscreen against HEK293T cells was carried out to assess hit compound selectivity for further prioritization. Mavelertinib (PF-06747775), a third-generation epidermal growth factor receptor tyrosine kinase inhibitor (EGFR-TKI), was identified as a potential new therapeutic based on indication, activity, and availability after reconfirmation. Mavelertinib has *in vitro* efficacy against metronidazole-resistant 713-M3 strains. Other EGFR-TKIs screened in follow-up assays exhibited insignificant inhibition of G. lamblia at 5 μM, suggesting that the primary molecular target of mavelertinib may have a different mechanistic binding mode from human EGFR-tyrosine kinase. Mavelertinib, dosed as low as 5 mg/kg of body weight or as high as 50 mg/kg, was efficacious in the acute murine *Giardia* infection model. These results suggest that mavelertinib merits consideration for repurposing and advancement to giardiasis clinical trials while its analogues are further developed.

## INTRODUCTION

Despite the enormous public health concern regarding the high prevalence (up to 30%) of giardiasis among malnourished children in economically challenged regions (1, 2), treatment of severe symptomatic diarrhea and stunting/poor developmental progression associated with asymptomatic giardiasis is often constrained by limited treatment options, marginal efficacy, and/or the development of resistance to available treatments (2). U.S. Food and Drug Administration-approved drugs with efficacy in human giardiasis include metronidazole, tinidazole, nitazoxanide, and furazolidone. A substantial number of clinical giardiasis cases are resistant to these nitro drug treatments (3, 4). Other potential therapeutics, like quinacrine, are effective in treating metronidazole-resistant giardiasis but have been withdrawn from the U.S. market due to poor tolerability (5–7). Even with increasing emphasis on new approaches to expand treatment options, development of new drugs against Giardia lamblia is limited (8). Given the public health need, accelerated development of alternative drugs for clinical use against all *Giardia* strains, including those with resistance to first-line treatments, is warranted. Recently described advances in G. lamblia phenotypic assays (9–11) and increased access to a large collection of well-defined pharmaceutical compound libraries provide new opportunities for identification and repurposing of inhibitors as treatments for giardiasis. The ReFRAME (Repurposing, Focused Rescue and Accelerated MEdchem) drug-repurposing library, a comprehensive collection of high-value compounds, is an example of advanced platforms for therapeutic discovery to support drug repurposing for neglected diseases (https://reframedb.org/) (12–17). This library is primarily composed of small molecules that are FDA approved, are undergoing clinical development, or have halted clinical development because clinical endpoints were not achieved. Screening annotated small molecule inhibitor libraries comprised of preclinical, clinical, or approved drugs can rapidly expedite the discovery of new medicines because hits can be quickly translated from phenotypic assays to proof-of-concept *in vivo* efficacy models (12–15). In this study, we screened the ReFRAME library to find potent G. lamblia-inhibiting molecules. The goal was to identify well-defined inhibitors possessing desirable physicochemical properties to accelerate preclinical development against sensitive and resistant strains. The screening effort identified mavelertinib (18) to be a potent inhibitor of G. lamblia growth and proliferation. Mavelertinib and other related third-generation epidermal growth factor receptor tyrosine kinase inhibitors (EGFR-TKIs) block the proliferation of small-cell lung cancer by covalently binding the reactive acrylamide group to a cysteine residue in the ATP-binding domain of mutant EGFR tyrosine kinase in these cells (18). Relative to other EGFR-TKIs, mavelertinib is more potent and has a favorable selectivity index for G. lamblia versus mammalian cell lines. Mavelertinib effectively clears *Giardia* infection in a mouse model at clinically relevant doses with no signs of toxicity, suggesting that it could be further developed for more effective treatment of giardiasis.

## RESULTS

### High-throughput screen of the ReFRAME library.

**FIG 1:**
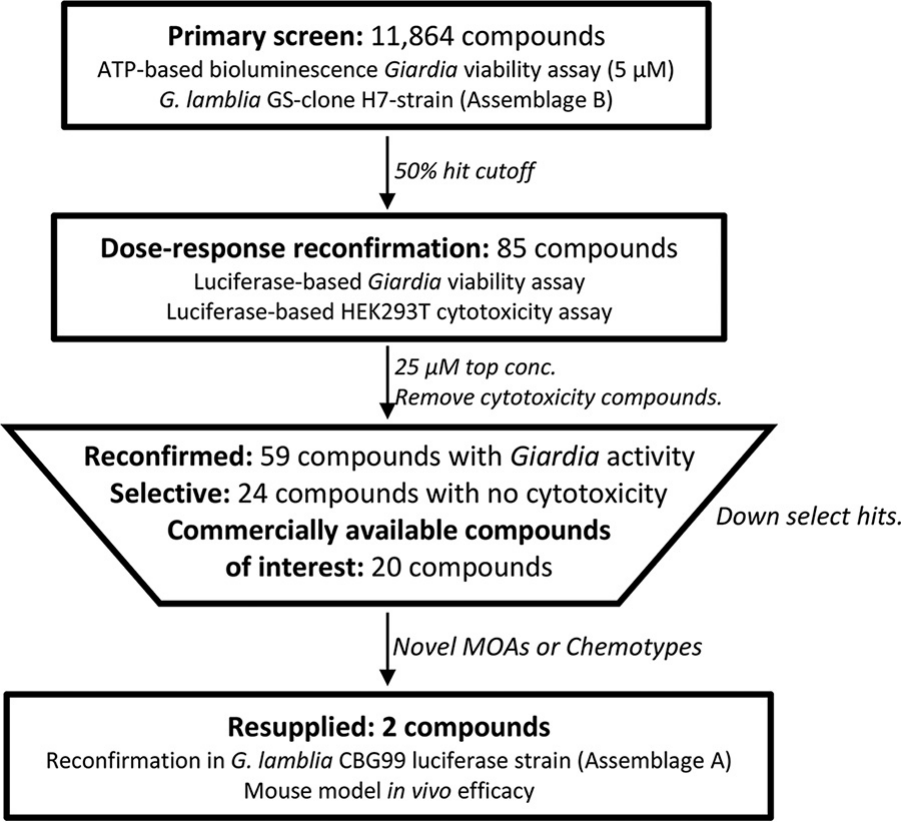
Flowchart of screening workflow and selection of high-value hits for resupply. The primary screening against G. lamblia GS clone H7 (assemblage B) was at a final concentration of 5 μM. The majority of the compounds that reconfirmed in dose response either were cytotoxic (29), had a chemical scaffold similar to the standard of care (18), or were unavailable for resupply (4), leaving only 2 selective compounds with a novel chemotype available for resupply. Based on reconfirmation in the G. lamblia CBG99 luciferase strain, mavelertinib and pelletierine were selected for resupply for testing in the mouse model of giardiasis.

The ReFRAME library was screened against G. lamblia GS clone H7 trophozoite growth by adapting a previously published high-throughput, ATP-based bioluminescence assay in a 1,536-well format (15, 19). Compounds were screened at 5 μM with metronidazole (40 μM) as an inhibitor control and dimethyl sulfoxide (DMSO) as a neutral control. A cutoff of 50% growth inhibition, as determined from normalized relative light units (RLU), was applied and resulted in 85 primary hits (0.72% hit rate; Z′ factor, 0.73). These primary hits were immediately assayed for reconfirmation and mammalian cytotoxicity against HEK293T cells in an 8-point dose-response format. Of the 85 primary hits, 57 reconfirmed anti-*Giardia* activity (half-maximal effective concentration [EC_50_], <25 μM) and 24 of the reconfirmed hits were selective (half-maximal cytotoxic concentration [CC_50_], >25 μM). Unsurprisingly, a large proportion of selective hits (18 of 24) included compounds currently in clinical use for giardiasis or those with similar chemical structures (Table 1), with only 6 having potentially novel mechanisms of action (MOAs) or new chemotypes for *Giardia*. The 6 hits were PR-104, CHF-6001, mavelertinib, propyl red, caroverine, and pelletierine. The overall workflow and down selection of hits are summarized in Fig. 1.

**TABLE 1:** Selective, reconfirmed hits that were available for resupply^*a*^

Name	Class	Additional comment	48-h Giardia GS clone H7 EC_50_ (μM)	72-h HEK293T cell CC_50_ (μM)	Selectivity index (CC_50_/EC_50_)
Azanidazole	Anti-infective	Azole; related to SOC	0.72 ± 0.06	>25	>35
Abunidazole	Anti-infective	Azole; related to SOC	1.08 ± 0.02	>25	>23
Ornidazole	Anti-infective	Azole; related to SOC	1.82 ± 0.04	>25	>14
Panidazole	Anti-infective	Azole; related to SOC	1.85 ± 0.68	>25	>14
Benzoyl metronidazole	Anti-infective	Azole; related to SOC	2.43 ± 0.23	>25	>10
Satranidazole	Anti-infective	Azole; related to SOC	2.49 ± 0.04	>25	>10
Carnidazole	Anti-infective	Azole; related to SOC	3.01 ± 0.06	>25	>8
Propenidazole	Anti-infective	Azole; related to SOC	2.55 ± 0.04	16.90 ± 0.05	>10
C-4	Anti-infective	Azole; related to SOC	5.23 ± 0.09	>25	>5
Tinidazole	Anti-infective; giardiasis SOC	Azole; related to SOC	5.7 ± 0.08	>25	>4
2-(5-Nitro-2-furyl) benzimidazole	Anti-infective	Azole; related to SOC	5.8 ± 0.13	>25	>4
Moxnidazole	Anti-infective	Azole; related to SOC	6.11 ± 0.11	>25	>4
Fexinidazole	Anti-infective	Azole; related to SOC	6.39 ± 0.16	>25	>4
Niridazole	Anti-infective	Azole; related to SOC	6.42 ± 0.06	>25	>4
Nitazoxanide	Anti-infective; giardiasis SOC	Azole; related to SOC	6.45 ± 0.11	>25	>4
Secnidazole	Anti-infective	Azole; related to SOC	6.57 ± 0.09	>25	>4
Aminitrozole	Anti-infective	Azole; related to SOC	7.11 ± 0.28	>25	>3
Metronidazole	Anti-infective; giardiasis SOC	Azole; related to SOC	7.12 ± 0.06	>25	>3
Mavelertinib (PF-06747775)^*b*^	Anticancer	Inhibitor of mutant EGFR-T790M; under investigation for non-small-cell lung cancer; highest clinical trial phase is phase II	2.34 ± 0.08	>25	>11
PR-104	Anticancer	Prodrug that is reduced to the hydroxylamine PR-104H, which can cross-link DNA in hypoxic tumor cells while sparing normoxic tissues; under investigation for both small-cell and non-small-cell lung cancer, liver cancer, and myeloid and lymphocytic leukemia; highest clinical trial phase is phase II	1.98 ± 0.07	>25	>13
CHF-6001	Respiratory illness	Inhibitor of PDE4; under investigation for chronic obstructive pulmonary disease and asthma; highest clinical trial phase is phase II	2.03 ± 0.1	>25	>12
Propyl red	pH indicator	Propyl red is a pH indicator dye that is structurally related to NSC240419, a compound previously under development as an anticancer therapeutic.	5.78 ± 0.07	>25	>4
Analogue of caroverine	Caroverine: muscle relaxer; tinnitus	This is an analogue of caroverine with two additional methyl ether groups on the benzene; information for this compound is unavailable. Caroverine is a calcium channel blocker approved for use in Austria and Switzerland as a smooth muscle relaxer and for treatment of tinnitus, and it is under investigation for people with loss of the sense of smell.	13.7 ± 0.1	>25	>2
Pelletierine^*b*^	Antihelmintic	Alkaloid derived from the root-bark of the pomegranate tree. This compound and related alkaloids have been shown to have antihelmintic activity, though the MOA is unknown.	20.30 ± 0.07	>25	>1

a Only two compounds were resupplied for follow-up testing based on the chemical scaffolds being distinct from standard of care (SOC).

b Compound resupplied for *in vivo* studies.

**FIG 2:**
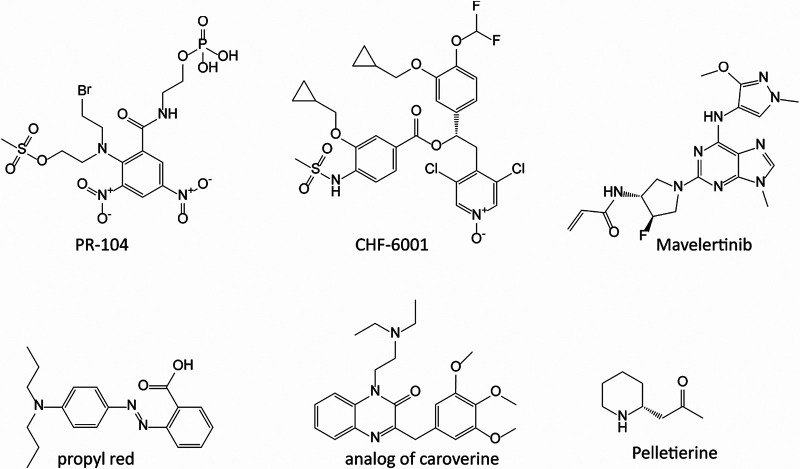
Chemical structure of selective hits with potential novel mechanism of action/chemotypes. The 6 primary hits, PR-104, CHF-6001, mavelertinib, propyl red, caroverine, and pelletierine, reconfirmed in follow-up assay have anti-*Giardia* EC_50_s of 1.98 μM, 2.03 μM, 2.34 μM, 5.78 μM, 13.70 μM, and 20.30 μM, respectively. Selectivity indices (defined as CC_50_/EC_50_ against G. lamblia GS clone H7 trophozoites versus toxicity in mammalian cell cultures) were >13, >12, >11, >4, >2, and >1 for PR-104, CHF-6001, mavelertinib, propyl red, caroverine, and pelletierine, respectively. The chemical structure of mavelertinib showing the reactive covalently binding acrylamide chemical functional group is shown.

The 6 novel hits (Fig. 2) ranged in potency from ~2 to 20 μM from various indications and uses (Table 1). Of these, the 3 most potent compounds (EC_50_s = 1.98, 2.34, and 2.03 μM) included the anticancer experimental therapeutics PR-104 (20) and mavelertinib (21) and the chronic obstructive pulmonary disease/asthma experimental compound CHF-6001 (22). Two other hits were propyl red and an analogue of the muscle relaxant and tinnitus therapeutic caroverine (23) (EC_50_s of 5.78 μM and 13.7 μM against *Giardia*, respectively). The sixth compound was pelletierine, an alkaloid found in the rootbark of the pomegranate tree (EC_50_ of 20.3 μM). Pelletierine and related punicine alkaloids have been used for their antihelmintic activity (24). Compounds for follow-up were selected based on commercial availability and chemical scaffold diversity relative to the standard-of-care drugs (metronidazole/nitazoxanide). Mavelertinib and pelletierine were resupplied for further studies.

### Reconfirmation and test against other EGFR-TKIs.

Mavelertinib was reconfirmed using the G. lamblia GS clone H7 (assemblage B) and G. lamblia CBG99 (assemblage A) strains, with resulting EC_50_ values of 2.34 and 0.15 μM, respectively (Table 2). Assemblages are subcategories of G. lamblia as defined by genomic differences and host variety. The host ranges for assemblages A and B include humans, cats, dogs, beavers, and guinea pigs, with other assemblages having more limited host specificity (25, 26). Subsequently, activity against G. lamblia WB6 (assemblage A) and metronidazole-resistant 713-M3 strains was investigated, with EC_50_ values of mavelertinib being 0.27 and 0.07 μM, respectively, while the EC_50_ value of pelletierine was >25 μM (Table 2) (11). The positive control, metronidazole, had a mean EC_50_ of 2.87 μM against the G. lamblia CBG99 strain. Mavelertinib is, therefore, at least 1.23- to 10-fold more effective against G. lamblia (depending on the strain) than metronidazole. Cytotoxicity assays of mavelertinib against CRL-8155 and HepG2 cells showed CC_50_ values of >80 μM (Table 2). Based on these results, pelletierine was deprioritized and the focus was shifted to mavelertinib.

**TABLE 2:** Results of EGFR-TKI cell growth inhibition assay against G. lamblia strains, cytotoxicity against CRL-8155 and HepG2 cells, and selectivity index

Compound	EC_50_ (μM) against indicated *G. lamblia* strain				CC_50_ (μM) against indicated cells		SI (CC_50_/EC_50_)^*a*^	
	GS clone 7	WB6	CBG99	713-M3	CRL-8155	HepG2	SI_CRL-8155_	SI_HepG2_
Mavelertinib	2.34 ± 0.08	0.27 ± 0.17	0.15 ± 0.10	0.07 ± 0.02	>80	>80	>34	>34
Pelletierine	20.30 ± 0.07	>25	>25	>25	ND^*b*^	ND	ND	ND
Metronidazole	7.12 ± 0.06	2.33 ± 0.06	2.87 ± 0.34	>25	ND	ND	ND	ND

a The ratio of efficacy of each inhibitor against G. lamblia GS-clone 7 trophozoites versus toxicity in mammalian cell cultures was used to calculate the selectivity index (SI), where the SI is defined as CC_50_/EC_50_.

b ND, not determined.

To investigate anti-*Giardia* activity with compounds similar to mavelertinib, 14 additional human EGFR-TKIs were sourced for screening against G. lamblia CBG99 at 5 μM. These included neratinib, rociletinib, AZD8931, afatinib, avitinib maleate, AG-490, lapatinib ditosylate, PD168393, PD153035-HCl, AG-18, dacomitinib, AG-1478, gefitinib, and erlotinib, representing different developmental generations with and without the potential for covalent binding. Unfortunately, none of the additional EGFR-TKIs inhibited G. lamblia growth above 50% at 5 μM, suggesting that the mechanism of inhibition of the molecular target in *Giardia* may be distinct from the mechanism used to inhibit human EGFR tyrosine kinase, and a targeted structure activity relationship study is warranted in the future.

### Pharmacokinetics (PK) and efficacy of mavelertinib in a mouse model of infection.

Mavelertinib was investigated for *in vivo* efficacy in an acute mouse infection model using the G. lamblia CBG99 strain (11). This strain is useful for noninvasive, quantitative measurement of antimicrobial treatment effects *in vivo*. The method relies on a stable constitutive reporter, CBG99, that correlates with total parasite load. Infection in female BALB/c or B6 gamma interferon (IFN-γ) knockout (KO) mice is clearly detectable with the IVIS *in vivo* imaging system (PerkinElmer) by 5 days postinfection and persists for more than 2 weeks (11). In the model, vehicle-treated mice have persistent infection, whereas successfully treated mice appear to clear the infection. Reliability of the model to measure *in vivo* efficacy was previously validated using methionyl-tRNA synthetase inhibitor compound 1717 (11). Compound 1717 was therefore used as a control along with metronidazole in the experiments reported here (11). Of note, metronidazole was administered at 20 mg/kg orally once per day (QD) for the treatment period, an insufficient regimen that will not clear the infection within the dosing timeline.

**FIG 3:**
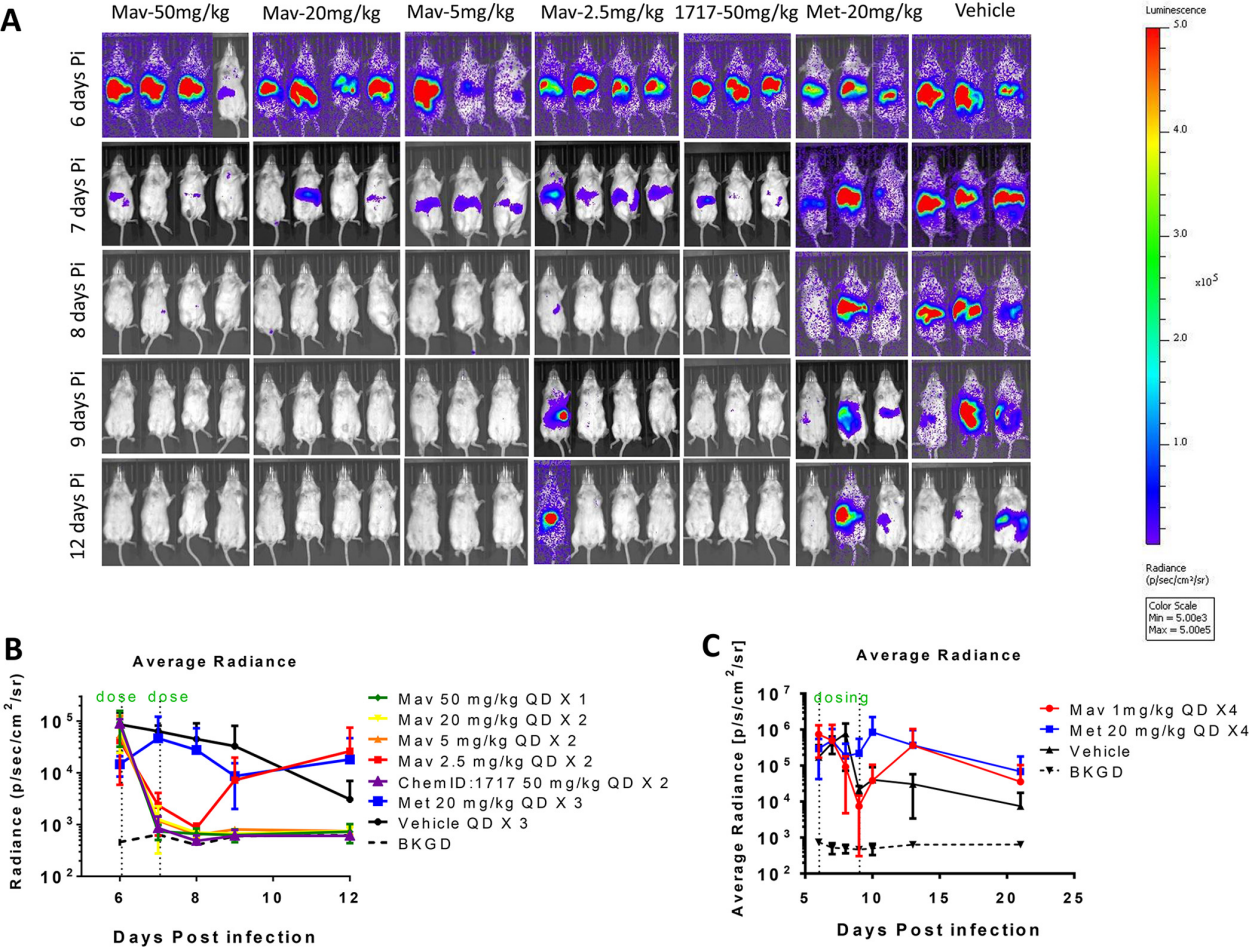
(A) Noninvasive imaging of G. lamblia CBG99 strain trophozoite growth in mice. Treatment started 6 days postinfection after confirmation of infection. Mice were treated with mavelertinib at 50 mg/kg × 1 (1 day), 20 mg/kg QD × 2 (2 days), 5 mg/kg QD × 2, and 2.5 mg/kg QD × 2. Metronidazole, dosed at 20 mg/kg QD × 3 (3 days), was used as a control. Positive controls included compound 1717 at 50 mg/kg QD × 2, while a group of mice dosed with the blank vehicle served as the untreated control. (B) Radiance plot and mouse images show absence of luminescence signal after a single 50 mg/kg treatment, 20 mg/kg QD × 2, and 5 mg/kg QD × 2 for mavelertinib as well as 5 mg/kg QD × 2 of compound 1717 relative to the untreated or metronidazole-treated controls. (C) Plot of the average measured photons (radiance) of bioluminescence of G. lamblia CBG99 trophozoites in mice treated with 1 mg/kg of mavelertinib and in untreated mice. In this experiment, the *in vivo* efficacy of mavelertinib (red plot) dosed at 1 mg/kg QD on days 6 to 9 postinfection in G. lamblia CBG99-infected B6 IFN-γ KO mice was measured. Treatment control for the study included 20 mg/kg of metronidazole (blue plot), vehicle (untreated) infected mice, and uninfected background controls (BKGD). Mavelertinib dosed at 1 mg/kg did not clear the infection after 4 doses.

Mavelertinib dosed at 50 mg/kg QD for 3 days appeared to clear the infection (data not shown). In subsequent experiments with 1- and 2-day dosing of mavelertinib at 50 mg/kg QD, all treated mice were cleared of infection and remained clear a week after dosing. Mavelertinib was further profiled in a dose-response experiment with the following regimens: 50 mg/kg QD for 1 day, 20 mg/kg QD for 2 days, 5 mg/kg QD for 2 days, and 2.5 mg/kg QD for 2 days. Except for the 2.5 mg/kg group, in which one of four mice remained infected, all regimens cleared the infection below the background signal (Fig. 3). A dosage regimen of 1 mg/kg QD for 4 days was subsequently tested for efficacy. None of the mice treated with this regimen were relieved of their infection (Fig. 3C). The lack of luminescence signals above background levels (Fig. 3A and B) about 1 week after the last of the therapeutic doses confirmed that parasites did not rebound after treatment with mavelertinib. Mavelertinib was therefore shown to be efficacious in a dose-dependent manner in an acute murine infection model, with 50 mg/kg QD administered once being as effective as some of the lower doses administered over longer periods. For compound 1717 treated control groups, 50 and 20 mg/kg QD for 2 days cleared the infection, whereas 5 mg/kg and 2.5 mg/kg QD for 3 days did not. In both of the latter cases, only one of three mice appeared to be cleared of infection (Fig. 3A; see also Fig. S1 in the supplemental material). The groups treated with metronidazole at 20 mg/kg QD for 3 days or the vehicle remained infected after treatment.

**FIG 4:**
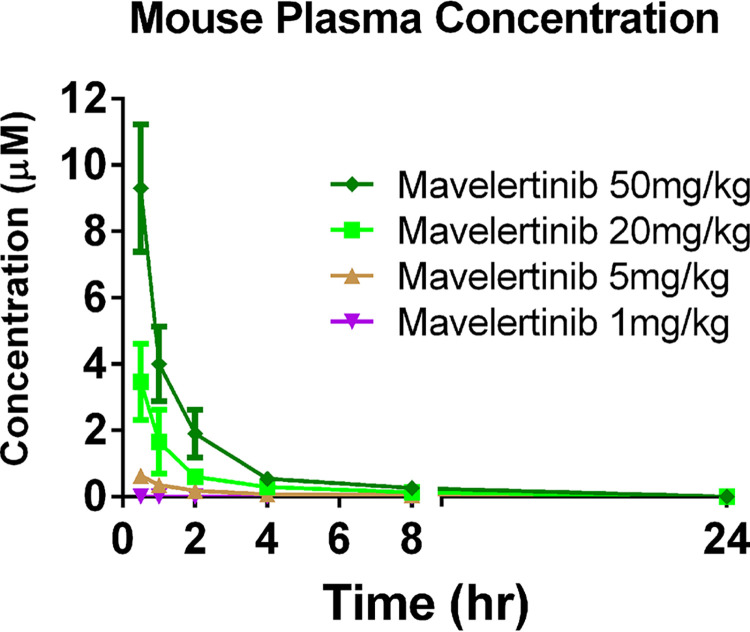
Pharmacokinetic analysis of blood at treatment concentrations. The time at which maximum plasma concentration (*C*_max_) was observed (*T*_max_) was 0.5 h in all cases. The 50 mg/kg dose had an average *C*_max_ of 9.3 μM at 0.5 h postdose. The 20 mg/kg and 5 mg/kg *C*_max_s were 3.5 μM and 0.63 μM, respectively. For the group treated at 1 mg/kg QD, mavelertinib was barely detectable in the plasma samples at all time points sampled. All dosage groups had plasma concentrations below the detection limit at 24 h postdose.

In all mavelertinib treatment groups, plasma trough levels taken on days 6 and 7 postinfection (24 h and 48 h after the first dose) were below the limit of quantitation. Single-dose mouse pharmacokinetic studies suggest that mavelertinib is cleared from plasma by 24 h (Fig. 4), which reflects what was seen in the *Giardia* efficacy experiments in which there was no accumulation of mavelertinib in the plasma. The 50 mg/kg dose had an area under the curve (AUC) of 812 min·μmol/L and an average maximum plasma concentration (*C*_max_) of 9.3 μM, with the average volume of blood plasma cleared of drug per unit time (clearance) being 0.0016 L/min. The 20 mg/kg and 5 mg/kg *C*_max_s were 3.5 μM and 0.63 μM, with AUCs of 300 and 89 min·μmol/L, respectively, and clearances of 0.004 L/min and 0.015 L/min, respectively. The time (*T*_max_) at which *C*_max_ was observed was 0.5 h in all cases, while average half-lives (*t*_1/2_) were 125.6, 174.8, and 239.2 min for the 50 mg/kg, 20 mg/kg, and 5 mg/kg doses, respectively. Mavelertinib, when dosed at 1 mg/kg QD, was barely detectable in the plasma of one mouse at 0.5 h postdose and was below the limit of detection at all subsequent time points. Clinical evaluation of the overall health of all mice used for *in vivo* PK and efficacy studies suggested no observable mavelertinib-induced toxicology potentials.

The observed systemic exposure may or may not be directly linked to *in vivo* efficacy, since we are unsure if mavelertinib was delivered to *Giardia* via the bloodstream or directly from the intestinal lumen. Hence, pharmacodynamic properties associated with *in vivo* efficacy could be pliable if toxicity to the host is minimal and exposure at the site of infection is sufficient for treating the disease. Even so, it appears that short courses of high doses may clear infection faster than low doses sustained over time for mavelertinib. The pharmacodynamics of this scaffold will be investigated further to ascertain whether intermittent high doses (*C*_max_) are better than low, consistent doses (time over EC_50_) in future studies of other analogues.

## DISCUSSION

In addition to the discussed limitations, including reduced efficacy and adverse side effects, many available giardiasis drugs also have broad-spectrum antimicrobial activity that may lead to the development of gut dysbiosis (27–29). Gut dysbiosis can complicate giardiasis treatment in malnourished children, a major population group for *Giardia* therapeutics. Given its high prevalence in resource-limited regions, giardiasis is a neglected infectious disease that could benefit from additional treatment options based on drug repurposing platforms (8, 11, 30) that will address these concerns.

Until recently, minimal effort has been dedicated to developing new anti-infectives against G. lamblia, despite the great clinical need. Renewed interest in giardiasis therapeutic discovery has spiked due to advances in G. lamblia reporter systems that are ideal for whole-cell high-throughput screens and *in vivo* screening of pharmaceutical libraries (8–11). The ReFRAME library could accelerate the process of finding new treatments against giardiasis and other infectious parasitic diseases afflicting millions in countries that have limited drug discovery and development resources (14–17). With 37 compounds from pharmaceutical companies approved for various human clinical uses and >250 currently in clinical trials (31), finding kinase inhibitors that could be readily repurposed for treating giardiasis and other indications among this pool is highly desirable. *Giardia* protein kinases are not specifically targeted by any available giardiasis drugs, making inhibitors of these enzymes attractive agents for development of therapeutics against sensitive and resistant strains (8, 30, 32). It should be noted that the data obtained in this study do not fully support a conclusion that the primary molecular target of mavelertinib in *Giardia* is a protein kinase, and more target identification work is needed. Nonetheless, selective inhibition will be required for antigiardiasis therapies irrespective of the molecular target.

Identifying inhibitors and optimizing drug candidates for selective inhibition of parasite targets is challenging due to high functional and structural similarities between many mammalian and parasite essential enzymes (33). This concern could significantly dampen enthusiasm for antigiardiasis drug development programs based on drug repurposing platforms. However, evolutionary drift that often results in subtle but essential differences in sequences or conformation may be exploited to achieve selectivity and clinical relevance (33–39). Mavelertinib belongs to a new generation of EGFR-TKIs that selectively target mutation of EGFR gatekeeper residue 790 (T790M) and other oncogenic mutations, thereby reducing safety risks. This may be the reason for its high selectivity index for *Giardia* versus mammalian cells and lack of adverse effects in mouse treatment experiments (18, 40, 41).

Research studies to decipher conformity or diversity in the degree of susceptibility to specific drugs among G. lamblia strains and assemblages have been vitiated by conflicting conclusions (26, 42). Nevertheless, the same classes of drugs are used to effectively treat all *Giardia*-associated diseases, which suggests a high level of homogeneity. Mavelertinib’s inhibitory effects on G. lamblia assemblages A and B as well as the metronidazole-resistant 713-M3 strain demonstrated here could therefore potentially be extended to other strains. Intriguingly, the primary molecular target in *Giardia* has yet to be defined. While knowledge of a well-defined primary molecular target is highly desirable, it may not be essential for product development in this case since mavelertinib is a repurposed agent with well-defined safety profiles (18, 43). Nonetheless, biochemical, genomic, and chemical-genetic studies, including selections of resistance, overexpression, RNA interference, and morpholino knockdown of protein expression to determine the target are ongoing (29, 44, 45).

Mavelertinib shows properties consistent with those of preclinical candidates for treatment of giardiasis and has already entered human Phase II clinical trials for lung cancer. Its chemical synthesis and safety profiles and a partial analysis of its pharmacokinetic properties have been described previously (18, 43). As part of mavelertinib’s Phase I clinical trials (NCT02349633), dose escalation studies in humans showed that administration of >150 mg with daily oral doses over 7 days in adults caused diarrhea and skin toxicities as the most common adverse events (43). Given that the average adult weight worldwide ranges from 57.7 kg in Asia to 80.7 kg in North America (46), a 150 mg dose equates to 2.6 to 1.9 mg/kg. Allometric scaling of the efficacious doses of 5 and 2.5 mg/kg in mice to the human-equivalent dose (0.081 × mouse dose in milligrams per kilogram) (47, 48) yields dose predictions of between 0.4 mg/kg and 0.2 mg/kg needed for average weight adult humans. This leaves a possible safety window of 4.6 to 13 times the efficacious dose, depending on the dose needed and the weight of the adult patient. Since the equivalent human efficacious dose (based on the murine efficacy data) is below the clinical trial threshold and the drug may not necessitate more than 7 daily doses, it could be speculated that all previously obtained good laboratory practices (GLP) toxicological data associated with the Phase I clinical trials remain relevant and may not need to be repeated. The direct implication for mavelertinib’s potential use as an antigiardiasis agent is that it can proceed to clinical trial Phase II, thereby fulfilling the goal of identifying and accelerating preclinical development of drugs against pathogens for which treatment options are limited or compromised by development of antimicrobial resistance. Reduced cost of development should have a direct impact on the cost of goods for the target population. This is certainly true for G. lamblia.

In the final analysis, a significant percentage of our population of interest is malnourished children, which underscores the need to investigate new leads for an improved safety index. Pfizer previously published detailed medicinal chemistry optimizations of the mavelertinib scaffold, along with several EGFR crystal structures in complex with mavelertinib and its analogues (18). This information could provide some guidance for further medicinal chemistry optimization to improve the therapeutic index, if needed. It is also possible that further optimization of mavelertinib may not be necessary with a carefully chosen dosing regimen.

## MATERIALS AND METHODS

### Compound library.

The ReFRAME library of 11,864 inhibitors was used for this study. Metronidazole (Sigma-Aldrich) and compound 1717 were included as controls (11). Resupplied compounds were purchased from vendors as high-quality powders. Mavelertinib was purchased from Sigma-Aldrich at ≥98% purity by high-performance liquid chromatography (HPLC). Neratinib, rociletinib, PD168393, dacomitinib, AZD8931, AG-18, AG-1478, PD153035-HCl, erlotinib, lapatinib ditosylate, AG-490, avitinib maleate, gefitinib, and afatinib were kind gifts from CSNpharm.

### Giardia lamblia culture.

G. lamblia WB6, G. lamblia CBG99, metronidazole-resistant strain 713-M3, and G. lamblia (Lambl) Alexeieff (GS clone H7; ATCC 50581) trophozoites used for the primary screening were maintained in modified TYI-S-33 medium (ATCC 2695) and assayed as described previously (49). Subsequent assays, including the mouse model of infection, used the G. lamblia click beetle green (CBG99) strain, which was grown and assayed as previously described (11, 49).

### Experimental compounds and high-throughput screening.

The ReFRAME library compounds (15) were assayed against axenic G. lamblia GS clone H7 trophozoites *in vitro*. The primary screen was conducted at 5 μM. Metronidazole was used at a final concentration of 40 μM as a control for G. lamblia inhibition, and dimethyl sulfoxide (DMSO) at an equivalent percentage (0.25%) was the negative control. All source plates were 384-well acoustic, transfer-compatible plates with compounds prediluted in DMSO at either 2 mM or 10 mM. For single-point testing, compounds were transferred into 1,536-well tissue culture-treated, white, solid-bottomed, high-base microwell plates (Corning; 9006BC) with an Echo 555 liquid handler (Labcyte) to a final concentration of 5 μM. For dose-response confirmatory testing, compounds were diluted 1:3 in an 8-point titration with a top concentration of either 3 μM or 25 μM (11). Primary screening was done with a single replicate, whereas dose-response testing was carried out in triplicate.

Compounds were prespotted into dry microtiter plates before 5 μL of 250 trophozoites/μL (1,250 trophozoites/well) was added with a MultiFlo FX multimode dispenser (Biotek). To prevent attachment to the bottle and tubing of the dispenser cassette, the bottle containing the culture was kept on ice throughout the dispensing process. Each assay plate was covered with a universal plastic lid (Greiner) and placed into a type A Bio-Bag (BD; number 261215). The bags were heat sealed and the anaerobic generator was activated according to the manufacturer’s instructions. After incubation at 37°C for 48 h, 5 μL of BacTiter-Glo (Promega) was added to each well and allowed to develop at room temperature for 5 to 10 min. Luminescence was read on a ViewLux uHTS microplate imager (PerkinElmer) with medium-sensitivity luminescence at 30 s of exposure.

### Mammalian cytotoxicity assays.

Mammalian cell lines were used for counterscreening for general cytotoxicity of hit compounds from the primary screen: human embryonic kidney cells (HEK293T; ATCC CRL-3216) were used in a luminescent ATP assay for detection of cell viability. Experimental details for the assays were described previously (50). Compounds in the follow-up assays were also tested for inhibition of CRL-8155 (human lymphocytic) cells and human hepatocellular carcinoma cells (HepG2; ATCC HB-8065) in a resazurin-based viability assay as indicators of potential host cell toxicity. Assays were performed as previously reported (51).

### Pharmacokinetic and *in vivo* studies of mavelertinib efficacy.

*In vitro* reconfirmation and expanded tests against EGFR-TKIs using the G. lamblia CBG99 strain were performed as previously described (11). Methods for pharmacokinetic studies and analysis of mouse plasma compound concentrations by liquid chromatography-tandem mass spectrometry (LC-MS/MS) have previously been described (34, 52, 53). The PK studies were performed in groups of 3 mice per compound. Each mouse received a single dose of 50, 20, 5, or 1 mg/kg of mavelertinib by oral gavage. Blood samples collected at intervals between 0.5 and 24 h were separated and extracted with acetonitrile for measurement of compound concentrations by LC/MS/MS. Mavelertinib was tested for efficacy in the luciferase reporter murine infection model using a noninvasive, whole-animal IVIS imaging method (11). Mice in an experimental group were dosed with 50, 20, 5, or 1 mg/kg of mavelertinib QD for 1 to 4 days. All animal experiments were approved by the Institutional Animal Care and Use Committees at the University of Washington.

### Data analysis.

Relative luminescence unit (RLU) values were analyzed in Genedata Screener (v13.0-Standard). RLU values were normalized to neutral controls minus inhibitors (DMSO-treated wells minus metronidazole-treated wells). For single-point primary assay plates, an additional run-wise median correction was applied to reduce screen artifacts, whereas no corrections were applied to triplicate dose-response plates. For the mammalian cytotoxicity assays, RLU values were uploaded into Genedata Screener, and the data were normalized to those for DMSO-treated wells minus puromycin-treated wells. A four-parameter, nonlinear regression curve fit (Smart Fit) was applied to dose-response data using Genedata to determine the half-maximal effective concentration (Giardia growth inhibition; EC_50_) or half-maximal cytotoxic concentration (mammalian cell growth; CC_50_) of each compound. Final filtered hits included those with an EC_50_ of ≤25 μM and a CC_50_ ≥10-fold greater than the EC_50_ value.
