# Supplementary material for: Repurposing the Kinase Inhibitor Mavelertinib for Giardiasis Therapy
Source: Antimicrob Agents Chemother. 2022 Jun 15;66(7):e00017-22. doi: 10.1128/aac.00017-22 (PMC9295539; doi:10.1128/aac.00017-22)
Supplement: Supplemental file 1 — Fig. S1. Download aac.00017-22-s0001.pdf, PDF file, 0.6 MB [file aac.00017-22-s0001.pdf]

Supplementary Fig 1

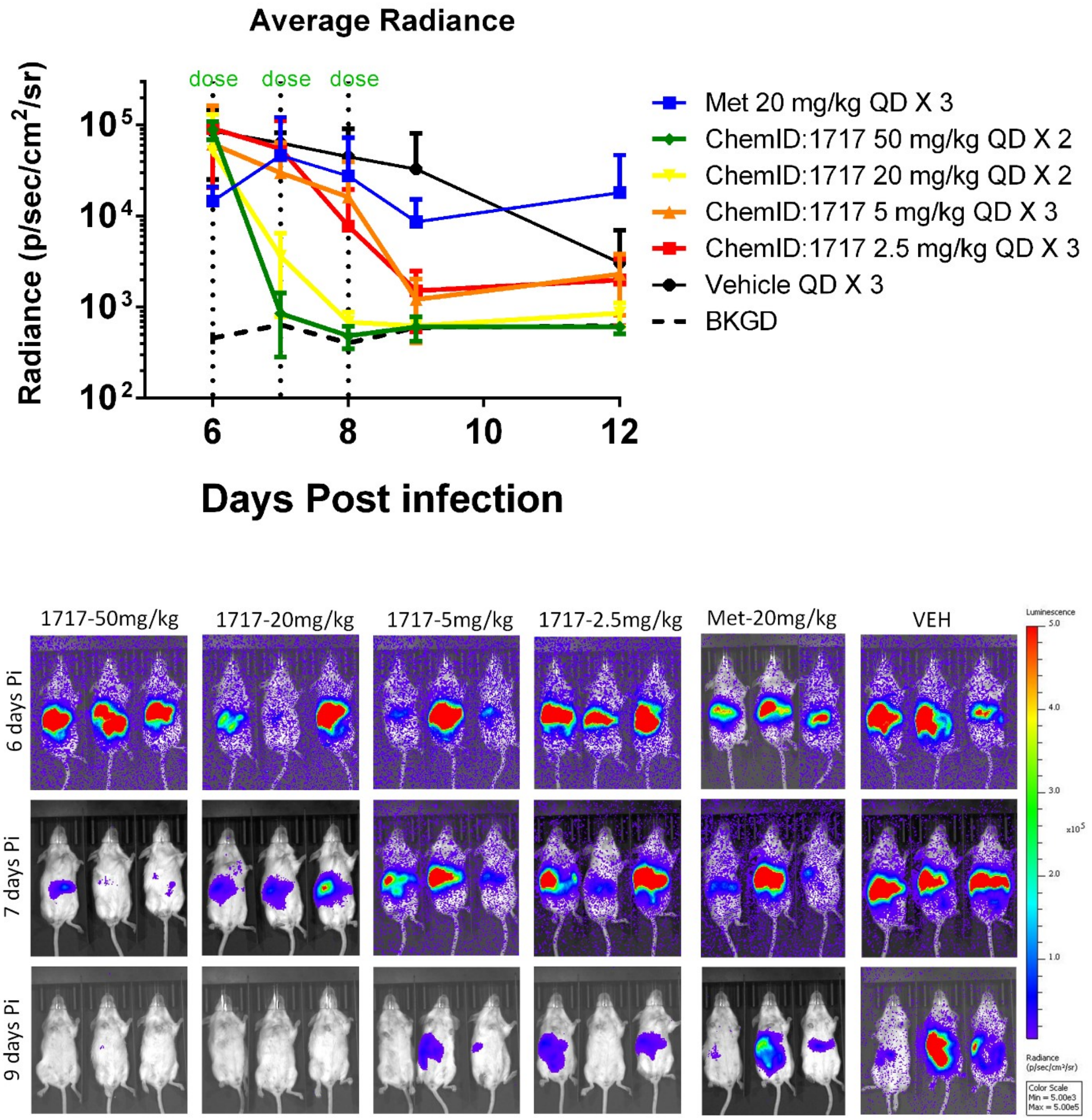

Fig S1: Radiance plot and mouse images before and after treatment with Compound-1717: Compound-1717 was dosed at 50 mg/kg x2 days, 20 mg/kg QD x2 days, 5 mg/kg QD x3 days, and 2.5 mg/kg QD x3 days. The infection was cleared in the 50 and 20 mg/kg QD x2 days groups, while the 5 mg/kg and 2.5 mg/kg QD x3 days did not.
